# The gendered health workforce: mixed methods analysis from four fragile and post-conflict contexts

**DOI:** 10.1093/heapol/czx102

**Published:** 2017-12-09

**Authors:** Sophie Witter, Justine Namakula, Haja Wurie, Yotamu Chirwa, Sovanarith So, Sreytouch Vong, Bandeth Ros, Stephen Buzuzi, Sally Theobald

**Affiliations:** 1 ReBUILD Consortium and Institute for Global Health and Development, Queen Margaret University, Edinburgh EH21 6UU, UK; 2 ReBUILD and Department of Health Policy, Planning and Management, Makerere School of Public Health, Kampala, Uganda; 3 ReBUILD Consortium and College of Medicine and Allied Health Sciences, University of Sierra Leone, Freetown, Sierra Leone; 4 ReBUILD and Biomedical Research and Training Institute, Harare, Zimbabwe; 5 ReBUILD and Cambodian Development Resource Institute, Phnom Penh, Cambodia; 6 ReBUILD and RinGS Consortia, Cambodian Development Resource Institute, Phnom Penh, Cambodia; 7 ReBUILD and RinGS Consortia, Cambodian Development Resource Institute, Phnom Penh, Cambodia; 8 ReBUILD and RinGS Consortia, Biomedical Research and Training Institute, Harare, Zimbabwe and; 9 ReBUILD and RinGS Consortia, Liverpool School of Tropical Medicine, Liverpool, UK

**Keywords:** Health workers, gender, post-conflict

## Abstract

It is well known that the health workforce composition is influenced by gender relations. However, little research has been done which examines the experiences of health workers through a gender lens, especially in fragile and post-conflict states. In these contexts, there may not only be opportunities to (re)shape occupational norms and responsibilities in the light of challenges in the health workforce, but also threats that put pressure on resources and undermine gender balance, diversity and gender responsive human resources for health (HRH). We present mixed method research on HRH in four fragile and post-conflict contexts (Sierra Leone, Zimbabwe, northern Uganda and Cambodia) with different histories to understand how gender influences the health workforce. We apply a gender analysis framework to explore access to resources, occupations, values, decision-making and power. We draw largely on life histories with male and female health workers to explore their lived experiences, but complement the analysis with evidence from surveys, document reviews, key informant interviews, human resource data and stakeholder mapping. Our findings shed light on patterns of employment: in all contexts women predominate in nursing and midwifery cadres, are under-represented in management positions and are clustered in lower paying positions. Gendered power relations shaped by caring responsibilities at the household level, affect attitudes to rural deployment and women in all contexts face challenges in accessing both pre- and in-service training. Coping strategies within conflict emerged as a key theme, with experiences here shaped by gender, poverty and household structure. Most HRH regulatory frameworks did not sufficiently address gender concerns. Unless these are proactively addressed post-crisis, health workforces will remain too few, poorly distributed and unable to meet the health needs of vulnerable populations. Practical steps need to be taken to identify gender barriers proactively and engage staff and communities on best approaches for change.

Key Messages

- In all contexts women predominate in nursing and midwifery cadres, are under-represented in management positions and are clustered in lower paying positions.
- Gender roles, shaped by caring responsibilities at the household level, affect attitudes to rural deployment and women in all contexts faced particular challenges in accessing both pre- and in-service training as compared to their male counterparts.
- Conflict and coping strategies within conflict emerged as a key theme, with strategies and experiences here shaped by gender, poverty and household structure.
- Most HRH regulatory frameworks did not sufficiently address gender. Unless these are proactively addressed post-crisis, health workforces will remain too few, poorly distributed and unable to meet the health needs of vulnerable populations. Practical steps need to be taken to identify gender barriers proactively and engage staff and communities on ways of addressing them.

## Introduction

Universal Health Coverage cannot be achieved at the global level if the issues of conflict and crisis-affected states are neglected (Witter 2015). Understanding the importance of human resources and the complexity of the context within which reconstruction takes place to achieve health sector redevelopment is crucial (World Health Organisation 2005). Issues of gender equality have featured strongly in the global post-2015 agenda for the Sustainable Development Goals (United Nations 2015), but have not necessarily been central to dialogue on health systems, universal health coverage or global health workforce shortages (Newman 2014). There is need for a stronger focus on gender across all aspects of the health system (Horton and Ceschia 2015) including human resources for health (HRH) (Standing 1997, George 2008, Newman 2014). This is arguably particularly critical in fragile and post-conflict contexts where HRH are often extremely limited and gender relations are in transition—in short, where there is an opportunity to build back better.

Fragile and post-conflict contexts typically face severe challenges recruiting adequate numbers of staff after the conflict or crisis, given that training institutions have been destroyed and large numbers of staff killed or fled (Witter *et al.* 2017b). Recruitment challenges are often made starker by a history of low, irregular remuneration for health professionals, which spans pre- and post-conflict. Achieving good staff distribution is challenged by loss of staff and flows to safer areas, and poor staff mix, unbalanced gender mix and absence of key health staff required at various levels of health facilities are often noted. Retention is also problematic, with internal (across sectors) and external brain–drain as a result of poor and irregular remuneration, poor HR management practices and limited promotion opportunities, alongside poor working conditions, especially in rural areas. Although there can be an influx of actors to support HRH reconstruction, these are frequently poorly coordinated, leading to distortions across areas, sectors and over time (Pavignani and Colombo 2009).

A growing body of wider evidence (Standing 1997, George 2008, Newman 2014) indicates that gender issues facing the global health workforce are significant and still under-prioritized in general, and for conflict and crisis affected settings in particular (Percival *et al.* 2014). Gender equality in HRH means that women and men have an equal chance of choosing a health occupation, developing the requisite skills and knowledge, being fairly paid, enjoying equal treatment and advancing in a career (Newman 2014). When gender inequalities and discrimination operate in the workforce outside of the awareness of HRH policy-makers, planners, educators and managers, they may impede entry into health occupations or contribute to attrition, absenteeism, lower productivity, poor health and low morale of health workers. The result is a limited pool of (possibly demotivated) formal and informal health workers to deal with today’s health and development challenges. However, in literature reviews recently conducted on human resource management (Roome *et al.* 2014) and health worker incentives (Witter *et al.* 2012) in post-conflict settings, gendered analysis of the health workforce and the issues faced in reconstructing it and achieving equitable access to quality essential health care were particularly absent.

By failing to accurately how gender shapes the functioning of HRH and describe the gendered nature of health work, women’s contributions to health systems continue to be unsupported as they are under-valued or not recognized at all (George 2008). There is clearly need to better understand the impact of gender discrimination in HRH (Newman 2014) and develop interventions to address gender inequalities in different contexts. The purpose of this article is to increase the evidence based on how gender relations affect HRH in post-conflict and fragile settings, which are too often neglected in health systems research and are critical to efforts to achieve UHC. Through presenting research conducted using comparable methodologies in four fragile and post-conflict contexts we aim to assess the implications of gendered HRH patterns for the rights and experiences of health workers and the opportunities and challenges these pose to the delivery of quality and equitable services and promoting universal health coverage.

## Methods

### Choice of contexts

Four countries were selected to represent different contexts and experiences of fragility and conflict within the ReBUILD research programme (Witter *et al.* 2017b): Zimbabwe, which continues to experience economic and political crises; Uganda (specifically the north where there has been confined conflict which emerged from the Lord’s Resistance Army-sponsored war in 2006); Sierra Leone, whose civil war ended in 2002 and whose Ebola epidemic (2014–16) brought further disruption to the country and health sector; and Cambodia, where a comprehensive peace post-Khmer Rouge began in 1999.

### Choice of methods

A mixed methods study was designed, using both retrospective and cross-sectional tools, summarized in Tables 1 and 2—for more details see Witter *et al.* (2012). The objective of the overall research was to understand changing health worker incentives and their policy implications in the post-conflict period. Most tools were used across all settings; however, some pragmatic adjustments were made (e.g. no survey was conducted in Cambodia as similar research had recently been conducted by another organization). Although gender was not the primary focus of the research, the research process and tools were gender sensitive and gender emerged as a key finding across the different methods deployed. Following this, two additional qualitative studies (both using the life history approach) and supported through the RinGs network^1^ were undertaken with gender relations as a primary focus: one on gender, HRH and leadership in Cambodia (Vong and Ros 2016, forthcoming), and a second on gender and HRH in rural Zimbabwe (Buzuzi *et al.* 2016).

**Table 1.** Summary of research sites and samples

	Cambodia	Sierra Leone	Uganda	Zimbabwe
Site selection	Six provinces (covering all four ecological regions)—one district from each, including urban, rural and those with more or less external support. The RinGS project was in one province, covering two operational districts	Four districts (covering all main regions)	Three districts in Acholi sub-region—most conflict-affected area	Two provinces—one well served and one under-served; three districts including urban, mixed and rural. The RinGs study was done in four districts in the Midlands Province
Sectors included	Public sector only	Public sector only	Public sector and private not-for-profit	Public (government, municipal and rural district council employees), mission and private sector
Timeframe	1979 onwards	2000 onwards (last phase of conflict; post-conflict since 2002)	2000 onwards (6 years during; 6 years after conflict)	1997 onward (economic crisis, and post-since 2009)
**Research tools and participant numbers (by gender, where relevant)**				
1. Stakeholder mapping		23 (7 f/16 m)	17 (3f/14m)	
2. Document review	59	57	59	76
3. Key informant interviews	19 (all male)	23 (10 f/13 m)	25 (12 f/13 m)	28 (13 f/15 m) RinGs *N*=11; 5f/6m
4. Life histories/in-depth interviews with health workers	19 (doctors, medical assistants, nurses, midwives) 14 f/5 m RinGS project: 20 (14f/6m)	23 (doctors, nurses, midwives, community health officers—CHOs) 12f/11m	26 (clinical officers, nurses, nursing assistants, midwives and others) 19f/7 m	34 (doctor, nurses, midwifes environmental health practitioners and clinical officers) 32 f/3 m RinGs study: *N*=19; 11f/8m
5. Quantitative analysis of routine data	√	√		√
6. Survey of health workers		310 (doctors, CHOs, nurses, environmental health officers. MCH Aides, Lab and pharmacy technicians) 178 f/132m		227 (doctors, clinical officers nurses, midwives, environmental health technicians) 127 f/100m; RinGs Study *N*=140 (nurses, midwives and environmental health technicians); 83f/57m

**Table 2.** Overview of research methods

Stakeholder mapping	The objective of this tool was to identify the key stakeholders who influence or are knowledgeable about HRH polices and their implementation. It was conducted in two countries as in Zimbabwe and Cambodia the topic was thought to be sensitive and not suited to group mapping. In Sierra Leone it was conducted at national level, whereas in Uganda, the exercise was done separately at national and district level. Male and female participants were drawn purposively from key constituencies (e.g. donors, Ministry of Health, Ministry of Finance, professional associations, NGOs, political stakeholders) and asked to brainstorm key stakeholders in HRH along two axes (influence and interest) and discuss changes through time
Document review	The objective of this tool was to describe the HRH policies, the reasons for their introduction, how they had been implemented, and any effects of the policy changes over the selected period, and the extent to which a focus on gender is included. The documents selected typically included national health strategic plans; national health workforce development plans; mid-term reviews of the health workforce development plans; health policy interventions on HRH, such as the incentive schemes; policies on remuneration (e.g. salaries, allowances, pensions, regulation of additional earnings); policy documents on recruitment (placement, promotion, retirement, and training of health workers); documents of organizations working in HRH; and academic studies or evaluations relating to health worker incentives. These documents were analysed using a thematic framework which was shared with the key informant interviews (KII)
Key informant interviews	Key informant interviews were undertaken to explore KI perceptions of health worker incentive policies, their evolution in the post conflict period, their implementation and effects. KIs from national down to local level were purposively selected, according to their knowledge of the focal topics. The interviews were semi-structured and focused on the following topics: Challenges for health worker attraction, retention, distribution and performance, post-conflict and at present and any differences for women and men health workers How policies had responded to these challenges Implementation experiences, constraints and lessons Their understanding of the effects of past policies Current thinking on reform options and priorities The interviews were tape recorded and noted after gaining permission from the participants. The interviews took place in a private place acceptable to the interviewee, such as their office. Thematic analysis using NVIVO (and ATLAS Ti in Uganda) was carried out on transcribed (and sometimes translated) texts. The analysis started from an agreed coding structure, shared with the document review, but with flexibility to alter according to the themes arising in the KII
Life histories (LH) with health workers	Life histories were deployed to explore health workers’ perceptions and experiences of their working environment, how it has evolved and factors which would encourage or discourage them from staying in post in remote areas and being productive. These were conducted with health workers meeting specific criteria (including gender, length of service in the area, to capture experiences of conflict and post-conflict periods) in selected health care facilities in the study areas using an open-ended topic guide. They were encouraged to produce visual aids, such as timelines. Life histories are arguably particularly conducive to gender analysis as participants are enabled to narrate in their own voices their experiences of work (and war or fragility) and how gender shaped their experiences (Ssali et al. 2016).The topic guide covered the following areas: How they became health workers Their career path since, and what influenced it, including the role of gender What motivates/discourages them to work in rural areas and across different sectors Challenges they face in their job and how they cope with them Conflict related challenges and how they coped Their career aspirations Their knowledge and perceptions of recent and current incentives. The life histories in the RinGs projects in Zimbabwe and Cambodia included a specific gender lens and included probes on issues relating to implementation of equal opportunities policies and legislation and how gender relations, expectations and norms at the household, organizational and socio-cultural levels affect health workers’ access to training, promotion and career advancement opportunities.The interviews and analysis followed the same procedures as for the KII.
Analysis of routine staffing data	The objective of this tool was to analyse trends in health worker availability, distribution, attrition, and performance during the post-conflict period. Existing human resource and selected service utilization data was collated from national, regional/district or facility sources (whichever were judged to be most reliable and complete). This was only completed for Cambodia and Sierra Leone; in Uganda, HRH data analysis was not included in the original study protocol, while in Zimbabwe it was included but not completed because of gaps in the HRH datasets. For the other two countries, data was collated for the defined periods using structured data extraction forms and analysed to describe the trends in health workers supply, distribution and output during the post-conflict period. In Cambodia, more extensive efficiency analysis was undertaken (Ensor, So et al. 2016)The indicators included numbers and trends over time for: staffing numbers for key cadres by gender and proportion of filled posts (where available); staff to population ratios; staff to output ratios; attrition rates (staff lost per year); and other relevant indicators, such as absenteeism
Health workers incentives survey	This was undertaken to understand the current incentive environment facing key health workers, their characteristics and the factors which motivate and demotivate them (to provide a quantitative measure to complement the analysis of the life histories). For this, a structured questionnaire was used to collect data from defined key cadres of health workers in face-to-face interviews. The study population included key cadres of health workers, with especial focus on those who are hard to retain. The sample size was based on the total number of workers in each category in the selected study areas, with a smaller proportion chosen for larger groups. Sampling was clustered by facility and non-random (small numbers available in each category and area meant that convenient sampling has to be used)The questionnaire focused on the following topics: Health worker characteristics Current earnings from different sources Current working patterns—public/private mix, other sources of income, dual practice, etc. Working hours and workload Perceptions of working environment and factors which motivate/demotivate and how these have changed over time Willingness to work or stay in rural areas The data were checked in the field, double entered, cleaned and analysed using SPSS or STATA software. Analysis was done according to cadre, region, type of facility, sector (in Zimbabwe) and gender

### Choice of analytical framework

In taking forward gender analysis we draw on relational theory which understands gender as multidimensional: ‘embracing at the same time economic relations, power relations and symbolic relations; and operating simultaneously at intrapersonal, interpersonal, institutional and society-wide levels’ (Connell 2009, Lorber 1994 cited in Connell 2012). This holistic multi-layered conceptualization is appropriate: health systems are not gender neutral; gender is a key social stratifier, which affects health system needs, experiences and outcomes and gender influences how people interact dynamically in complex, multi-faceted and context-specific ways, reflecting varying interests, values and power (Morgan *et al.* 2016). The literature on gender and HRH illustrates the complex relational nature of gender and how this can shape health worker experiences, organizational norms and cultures and the responsiveness and resilience of health systems through time and space. Hence, the occupations commonly performed by women and men reflect and reconstitute inequitable gender relations, but the way this occurs in different contexts is poorly understood, with particular evidence gaps in post conflict contexts.

Overtime frameworks have identified how gender norms, beliefs, roles, time allocation, division of labour, access to resources and rules and decision making constitute gender power relations which drive inequality at organizational, community and individual levels. We use a gender framework developed by Morgan *et al.* (2016), which was developed following review of 42 gender frameworks (15 of which focused on health) to enable a holistic and relational approach to analysing the multiple ways and levels in which gender relations shape health systems. The framework includes key domains which constitute gendered power relations: access to resources, including education, skills, information, income, employment, etc. (who has what?); division of labour within and beyond the household (who does what?); social norms, ideologies, beliefs and perceptions (how are values defined?); and, rules and decision making (who decides?). The framework is used to assess what constitutes gender power relations in relation to health workforce patterns, norms, values and decision-making processes. Key to a relational understanding of gender is the notion that gender is dynamic, context-specific and amenable to change; hence, the framework also includes domains related to how gendered power is negotiated and changed through time and space. Although the framework breaks down different (often overlapping) concepts and categories to support analysis, its application should ideally enable a multi-layered, holistic and relational understanding of gender and its impact (in this case on HRH within fragile and conflict affected contexts), as well as strategies for change.

### Ethics

Ethical approval for the research was obtained from the relevant national ethical committees in the four countries, as well as at the central institution. Precautions were undertaken to obtain informed consent, to assure confidentiality of information, anonymity of respondents, to undertake research in a sensitive manner and to keep data secure. This research topic dealt with sensitive issues relating to pay, gendered experiences, progression (or lack of) and health worker behaviour. Research locations were carefully selected to ensure privacy and all data were anonymized.

## Results

Table 3 lists how we have analysed the datasets against the different domains of the gender analysis framework, looking for patterns across the different settings and being mindful of the very different post conflict contexts studied.

**Table 3.** Gender analysis framework used

Domains and questions		Interpretation and sources of data in this study
*What constitutes gendered power relations*		
Who has what	Access to resources (education, information, skills, income, employment, services, benefits, time, space, social capital etc.)	patterns of employment (based on data and document analysis); access to pre-service and in-service training (from document review and in-depth interviews); differential incomes (from survey and document review)
Who does what	Division of labour within and beyond the household and everyday practices	distribution across areas, cadres and sectors (from documents and LHs); juggling productive and reproductive work (from LHs)
How are values defined	Social norms, ideologies, beliefs and perceptions	factors underlying motivation to join, career choices, motivation and experiences of policies (from LHs)
Who decides	Rules and decision-making (both formal and informal)	career choices through time experiences and opportunities for management (from LHs and KII); LH
*How power is negotiated and changed*		
Individual/people	Critical consciousness, acknowledgement/lack of acknowledgement, agency/apathy, interests, historical and lived experiences, resistance or violence	perceptions of justice & coping strategies when faced with conflict and crisis (from LHs)LH(from LHs)
Structural/environment	Legal and policy status, institutionalization within planning and programs, funding, accountability mechanisms	HRH policies on gender (document reviews & KII); policy and practice (all sources)

Source: Adapted from Morgan et al. (2016)

### Who has what?

#### Patterns of employment

The health workforce in post-conflict areas, like other settings, reflects a strongly gendered pattern, with a preponderance of women employed in mid- and lower-level cadres. This was echoed in our research samples—with a higher ratio of women to men interviewed in the health worker life histories, but a higher ratio of men interviewed in the key informant interviews (which focused on managers), across all settings but especially in Cambodia, where all key informants were male.

For example, in northern Uganda, the health workers interviewed were 77% female and were mid-level cadres. This was not a sampling choice but reflected the staffing situation in the conflict-affected districts, where those who had stayed during the war and continued to provide front-line health care post-conflict were predominantly female. It might have been expected that during a conflict, the gender balance of the workforce might change, with fewer women prepared to expose themselves to the dangers of rebel forces, but this was not the situation on the ground (Namakula and Witter 2014a, b).

**Figure 1.**
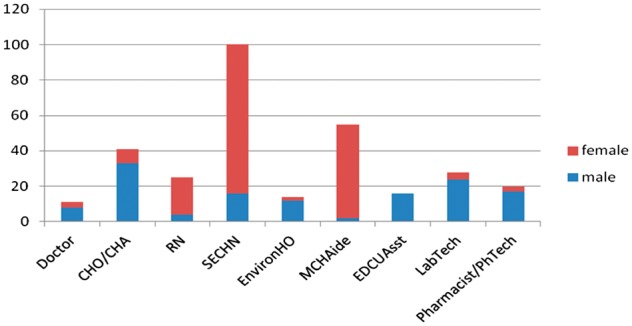
Gender of health staff surveyed in Sierra Leone. Source: Witter *et al.* (2014).

The gender imbalance between cadres is also demonstrated by our Sierra Leone survey, in which registered nurses (RN) and state-enrolled community health nurses (SECHNs) and Mother and Child Health Aides (MCH aides) are predominantly females, while medical doctors, Community Health Officers (CHO) and Aides and technicians are nearly all male ( Figure 1). In Sierra Leone, the nursing profession is seen as a predominately female career pathway, with male nurses reporting sociocultural difficulties in being a nurse (Wurie and Witter 2014).

Zimbabwe’s health system fits the same pattern, with the nursing and midwifery specializations dominated by women, compared with the medical and environmental health professions. This arrangement was typical of all three sectors studied (municipal, public and mission) and all three districts included in the analysis. These patterns were explained in part through gender stereotypes which constructed females as better at providing care than males: ‘*Male nurses were difficult and inefficient, while female nurses are efficient and a marvel to work with*’ *(Male KII, Zimbabwe*)

The same gendered patterns were also replicated in Cambodia where a discrete choice experiment conducted in 2010 found that two-thirds of nursing students were female; all midwifery students were female and two-thirds of medical students were male (Bundeth *et al.* 2011).

#### Access to training

Across the four settings, access to training—especially in-service training and upgrading—was found to be problematic for women in particular. As training often required time away from home it was difficult for health workers in more isolated areas, particularly women, to attend. In Uganda, the effects of the conflict, plus household caring responsibilities, made it hard for health workers to upgrade their skills (Namakula and Witter 2014a, b).

‘*I got distinction in all my papers, but unfortunately up to today I have not gone for registration because I have a lot of responsibilities, we have many orphans who were lost by the rebels, so with the little money I’m trying to push them ahead to study*’ (Female IDI, Uganda)

The female participants who managed to access further training, particularly the trainings that lasted for longer periods such as 1–2 years, were always worried about how their children were surviving back home in challenging contexts.

*‘… During registration life was a bit difficult in the sense that of course you have left a family behind. You left young kids behind so I could think about that kid and I had other children. …I had lost my husband in 1997 so I immediately came back from training*’ (Female IDI, Uganda)

This issue was also highlighted in Cambodia, where female participants faced challenges in child care for longer periods of training, although some sympathetic managers were able to negotiate other routes to career development for their committed female staff (So and Witter 2015):

*‘…Majority of my staffs, especially female staffs, could not go for a long period of formal trainings because they had to take care of their family; but they showed a strong commitment to stay working with us. For rewarding or promoting them, we often assigned staff with outstanding performance to take management role of health development programme or take partnership with NGO programme. So that they could get salary supplement for increasing their income and get opportunity to attend the training courses offered by those NGO programme…*’ *[(Male KI, Cambodia)*

In Sierra Leone, further training opportunities were motivating for all staff, but it was noted that the location of training differed for men and women, with men more likely to benefit from international training (compared with women, who tended to be trained nationally or in the West Africa region) (Wurie and Witter 2014). This may link to differential levels of seniority, shaped by gender relations.

Access to training in Zimbabwe is based on seniority and years in service. The system was perceived by the participants/health workers to be transparent (Buzuzi *et al.* 2016). However, men tend to be ‘impatient’ with the system and opted for self-funding training courses. In contrast, most women waited their turn and in addition were sometimes unable to take up training opportunities due to gendered family responsibilities. In terms of relocation for career development or new opportunities a clear norm emerged of wives following husbands, meaning women resigned from their jobs to seek new ones, therefore sacrificing the accruing of years of service required to access training and the opportunity for promotion. Human resource managers prefer to deploy men to very rural areas as they believe they will stay longer and not request transfers. Rural posting was discussed positively as a strategy to gain a wide range of experiences (in the absence of senior medical staff) and in turn was valued (by men in particular who faced fewer family constraints to moving) in terms of future access to training, invitation to international workshops and promotion.

#### Differential income

Although we did not systematically investigate pay differences, the gendered pattern noted above has implications for female health workers’ incomes. Across all contexts investigated gender relations and occupational norms meant that women were clustered in lower paid posts. In addition, there may be differences in additional sources of revenue. For example, a study in Cambodia of 2708 health staff in 2013 found not only that male salaries were significantly higher, but also that dual practice income was on average twice as high for men, resulting in an overall average monthly earning of USD$676 for men, compared with $436 for women. 63% of male income came from dual practice, compared with 47% for women (HR Inc 2013). This presumably reflects higher earning opportunities for doctors, as well as urban/rural distribution patterns.

In Zimbabwe, the most glaring difference in incomes was sectoral rather than gender-based. Municipal salaries tend to be higher for all cadres, as the municipalities have sources of income (such as local taxes) which allow them to top up the meagre official salaries (Chirwa *et al.* 2016b). The distribution of staff across cadres and sectors therefore determines public pay. Additional income is also important, though, with more of the female cadres indicating that they were involved in additional non-health care related income-generating activities.

In Sierra Leone, in the health worker survey, male counterparts tended to have a higher earning power compared with females in the same role. However, this was only significant for Community Health Officers/Aides, which may reflect levels of seniority in this male-dominated cadre (Witter *et al.* 2014).

#### Who does what and where? How gender shapes distribution of staff across contexts and though time

Distribution of staff is a key challenge for many countries, including post-conflict ones—even where numbers of staff are adequate for some categories in some countries, distribution to rural areas is a near-universal challenge (McPake *et al.* 2013). It is interesting to consider how gender interacts with other factors which drive a preference for urban areas (such as the increased opportunities for additional earnings, but also opportunities for children to study, etc.). A discrete choice conducted in Cambodia in 2010 found that gender was not an important driver of location preference, but that female medical doctor students had a higher propensity for rural service than their male colleagues, perhaps for family reasons (Bundeth *et al.* 2011). However, KIIs explained that women face particular challenges in being located in rural areas (So and Witter 2015):

*‘…Some raised poor accommodation with relatives while some complain about it’s hard for them to stay away from their family because they are female….’(male KI, Cambodia)*

Retention of newly recruited health workers is also a problematic issue, especially for female workers in the least developed area or where there are bad road connections. It was echoed by all health mangers consulted that:

*‘…It was very difficult for us as the PHD [provincial health department] managers to keep secondary midwives in the newly established health centres located in the poor districts. Many often try their best to get support from high ranking official for transferring them to work in the HC or RH of their own choice…’(male KI, Cambodia)*

Alongside rural–urban differentials in the Cambodian context, there are important differences post-conflict between Khmer Rouge and government-controlled districts. Mine-fields continue to pose risks for health workers in former Khmer Rouge zones, which are also associated, especially for women, with fear of violence (KII report). Nonetheless, those with family and marriage ties, as well as local businesses, are drawn to serve in those areas.

In Zimbabwe, men were more likely to be posted to rural areas, which was seen as beneficial for promotion. In Sierra Leone, separation from family was predominately expressed as a demotivating factor by female health workers in rural postings. These health workers based in the hard to reach areas are faced with a number of constraints on the job, such as difficult terrain, bad roads, poor communication, delayed allowances or no allowances, whilst having to run two households and deal with an additional emotional burden of being separated from their families (Wurie et al. 2016b). Gendered power relationships mean that women are less likely to be able to take their family with them if they are posted away from home. This poses an emotional strain on these female health workers, who in addition, like their Cambodian counterparts, expressed concerns about personal security:

*‘Whenever you have to travel you have to go by commercial vehicle and that one is risky, leaving your family back home, being in the provinces so long, you are away from your family it has created some sort of social isolation and stress. Any way it is too much’(Female, LH, Sierra Leone)*

Reproductive realities also shape health worker experience and deployment and this emerged as an area of concern for KII in northern Uganda (Namakula and Witter 2014a, b). In the context of understaffed facilities, there is limited ability to cover for maternity leave, which means that occupations which are occupied by women are seen as vulnerable.

*‘… but midwives are few … because in practice, all health centre IIIs and IVs should have a midwife. However, you find that we have only one midwife in each of those levels and if she is on [maternity] leave, there is a big gap because there will be no one to remain. And then the HCIIs, with the new construction system, they have a maternity attached but you find that there is no midwife in those facilities. Those are the only challenging part on human resources’(male KI, Uganda)*

## Values and motivation

The life histories explored motivation to join the professions across the four settings. Although there were common elements, there were also differences of emphasis between men and women, which may in part reflect the interplay between gendered power relations and the realities of context. For example, in Zimbabwe, women were more likely to cite passion and a calling as motivation to join the nursing profession. For males, life circumstances such as needing to support family or entering training because of lack of fees for schooling were more likely to be cited (Chirwa *et al.* 2016a).

In Uganda, the research highlighted the desire for professional status, particularly the wish to wear a uniform, attracting staff to join the medical profession. Having an ‘Innate caring personality’ was cited more frequently by females than males. Males mostly joined because of a ‘calling’ or were motivated to join from external sources (Namakula *et al.* 2013).

*‘[…] since child hood, I had so much sympathy for sick people and I could care for any one even before I was trained. […]I have had that heart since I was* born’ (Female LH, Uganda)

In Sierra Leone, family, including influential family members and financial situations, played a role in the decision to join the health profession. Female health workers were predominately influenced by members of their families to join the health profession, which was not as evident for males (Wurie and Witter 2014).

‘My father … well I think his friend was related to the then principal of the school … so he encouraged him that one of the children … should do nursing and I decided to offer myself yeah’ (Female LH, Sierra Leone)

Both male and female health workers in Sierra Leone cited lack of financial support as a factor, though the routes chosen varied, with men more likely to choose the paramedical school and women the nursing school, both of which used to offer tuition-free courses.

## Decision-making

### Career choices through time

As health workers progressed in their career, their needs and expectations changed. Staff were more likely to notice and respond to differences in pay and restrictions on earnings across institutions and sectors, especially when children reached secondary school, which is a more expensive time in the life-cycle in all four settings. In Uganda, married female health workers seemed better off than their colleagues in female-headed households, given that they tended to receive some assistance from their husbands (Namakula *et al.* 2013):

‘… during that time (2006) … I worked for six months without payment… but my husband was assisting me…he was in Sudan …working with the NGOs. When I finally got salary, it was only 227 000 [Ush – about $66]. I had to use it just for feeding the family. With the school fees and the rest my husband used to do it because my money was too little’ (Female LH, Uganda)

During their active workforce stage, health workers changed jobs and migrated/moved from one place to another. Although both female and male health workers requested for a transfer in order to go back and be with their families (who were in other districts or in a rural part of the district in which they were working), requests for transfer to join family was more common among females in order to be closer to their families or carry out further productive and reproductive work.

In Zimbabwe, we found that age affects motivation, with older nurses and midwives identifying passion and salary as both important factors while younger cadres were motivated by salary and security of the job (Chirwa *et al.* 2016b).

### Experiences and opportunities for management

As a result of the different factors outlined above, women are under-represented in management in all settings. For example, a research project sampling in eight districts of Uganda found that men occupied 77% of senior management jobs in health, while 63% of middle management jobs were occupied by men (Newman 2013).This does not reflect the overall staffing situation: in public service as a whole in Uganda, 67% of employees are male and 33% female. In Cambodia too, managers in the health sector are mainly men (87%, according to a 2011 report^2^); in life histories, women who progressed to leadership levels emphasized the strong family/parents and husband support in their career, or were single or married late. When offered leadership positions, women tended to take a more consensual approach, seeking approval from their families first; men were perceived as more confident to push forward in their career compared to women (Vong and Ros 2016, forthcoming).

Similarly, in Zimbabwe, while the nursing profession is dominated by women, the higher levels of managerial posts, such as Community Nursing Officer (CNO) and District Nursing Officer (DNO), tend to be occupied by men. There were more women however in the matron and sister in charge posts in our study districts, across all sectors. The reason for the highest managerial posts being occupied by men is not clear; however, some key informants alluded to male preference being linked to the responsibilities of the posts which require constant travel to monitor health facilities in the districts, including the most remote facilities, usually linked with very bad roads. Furthermore, the post-holders are responsible for moving health workers to different posts at times to deal with sudden vacancies that have occurred. Men were seen by senior managers as more suitable for these tasks (Buzuzi *et al.* 2016).

There have not been any attempts to consider nurses and other professional categories as being able to provide leadership in health delivery in Zimbabwe. The position of District Medical Officer (DMO) is reserved for a doctor, which with current employment patterns favours men. Recently, there has been affirmative action to allow more females to train as doctors, but it will be some years before these young doctors gain adequate experience to be able to compete for promotion and appointment to DMOs. These regulations also make it even more difficult to imagine a nurse or midwife being promoted to the post of DMO. Most nurses and midwives aspire to be promoted to sisters in charge, matrons, CNOs, DNOs and Provincial Nursing Officers.

## Negotiating and changing power

### Lived experiences, perceptions of justice and coping strategies during crisis and conflict

Participants in our study were affected by economic crises, as well as conflict. In Zimbabwe, the crisis period affected the health sector with respect to staff motivation and performance. Irregular attendance, moonlighting and selling various wares during working hours increased, affecting health service delivery (Chirwa *et al.* 2016a). These were coping mechanisms to deal with household economic problems caused by the crisis. Most of the female cadres in the nursing field indicated that they participated in these activities in order to survive. Indeed the few nurses and midwives who remained at health facilities in the underserved areas were able to do so because of the income they got from these economic activities. Both female and male cadres in rural areas also indicated that they ventured into agricultural activities to supplement dwindling incomes from formal health care work. The crisis eroded incomes such that meeting financial obligations like school fees for children was no longer possible and hence it was imperative that sources of additional income be sought. Health workers in the urban areas tended to sell various wares while in the rural areas crop production and poultry and cattle rearing were the most common economic activities.

There were similar coping mechanisms in relation to low salary for both female and male health workers in Uganda (Namakula *et al.* 2013). Respondents coped by carefully managing their resources; some went into agriculture or opened up side enterprises such as drug shops, secretarial bureaus and kiosks, whereas others would have to undergo family separation to work in different jobs.

Conflict was a major contextual factor which affected both the lives and career choices of health workers. Participants recalled traumatic situations and coping strategies during conflict, as well as stressing their commitment and resilience. Strategies for coping with the conflict included task shifting, disguising themselves, hiding amongst the community and finding innovative ways to work with limited supplies (Namakula and Witter 2014a, b).

*‘…the rebels came, abducted the in-charge and killed a nursing aide. I managed to escape but … I ran among the community members… I would not treat my hair… they [rebels] would follow you because you look different from other people. … That is why they [rebels] did not focus on me particularly because I was exactly like the community. And I used to buy simple clothes for my baby like for the community, even this one*^3^*’(Female LH, Uganda)*

They also deployed psychological strategies such as fatalism and relying on their faith. Different responses by men and women might have been expected but analysis of interviews did not reveal any strong gender differences. Those who stayed, male and female, were able to find internal and external resources to help them to cope with extreme risks of death and abduction, as well as more mundane challenges of poor working conditions. However, unlike their male counterparts, women had to combine motherhood and reproductive roles and responsibilities with health work during the difficult context of the conflict.

*‘In Adilang …I remember struggling to help a woman, kneeling with no bed but just on the floor, so that was the worst experience I had. I was also pregnant and I got a miscarriage’ (Female LH, Uganda)*

In Cambodia, life histories revealed that during conflict health workers in high-risk areas had different gendered coping strategies: male health workers train themselves to use weapons for protection, whereas female health workers found ways to escape (So *et al.* 2016). In Sierra Leone, health workers were able to talk not just about conflict but also the Ebola crisis. Health workers in Sierra Leone were victims in both. In the former, health workers were targeted for kidnapping to provide health services behind rebel lines (Wurie and Witter 2014). Female health workers also faced the additional risk of sexual violence if kidnapped by the rebels.

During the Ebola outbreak in 2014–15, women predominated amongst the lowest cadres of health workers who were been critical to the Ebola response—including volunteers, traditional birth attendants, community-based motivators and community health workers. Thus, gender norms shaped vulnerability to Ebola. Men on the other hand were more involved in burial rites, putting them also at risk. During the Ebola crisis, health workers reported different pressures and methods of coping. In some instances, female health workers were prohibited from working by their male partners and relatives. Female health workers reported religion and peer support as coping mechanisms during the outbreak. Men in general reported being motivated by the sheer urgency of having to curtail the spread of the virus (Wurie *et al.* 2016a).

### The role of regulatory frameworks in addressing gender relations

In most settings, policy and regulatory frameworks and implementation practices are gender-neutral, not sufficiently recognizing the different pressures and challenges facing different groups of male and female health workers. In Zimbabwe, for example, all policies on remuneration, retention, training and promotion are gender neutral and all health workers are governed by the same rules and regulations. The only exception is maternity leave. In addition, given the current preponderance of male environmental health professionals, there are provisions to have 10% training slots reserved for females. No preferential treatment for men who intend to pursue careers in the nursing profession has been put in place, because (according to one KII) of the expense of having to redefine current residential arrangements at training colleges (Chirwa *et al.* 2015).

In Sierra Leone, despite some references to gender mainstreaming in donor documents, such as the WHO Country Cooperation Strategy (2008–13), there is no policy commitment to address gender imbalances in the health sector in national plans or post-Ebola recovery plans within the health sector; and links have not been made with the gender and Ebola plan developed by the Ministry of Gender, Youth and Community services.^4^

Analysis of all main policies for HRH and related studies in the past 15 years in Uganda revealed a marked absence of gender analysis (Ssali *et al.* 2016). Problems of recruitment, retention, distribution and performance receive considerable attention, with particular focus on hard-to-staff areas and also particular cadres who are in shortage, but there is no evidence of gender analysis or understanding that gender may play a role. The Ministry of Public Service produced guidelines for gender mainstreaming in 2011. However, despite its emphasis on women reaching senior positions, gender sensitive language, gender focal persons and gender-disaggregated data, we found little evidence of these being reflected in the health sector. Only in Cambodia did our research findings indicate that through time the national and provincial government structures have become more gender-sensitive, with the implementation of gender focal points and constitution of gender mainstreaming action groups. These work to develop Gender Mainstreaming Action Plans and ensure their implementation and monitoring. They also collaborate with the Ministry of Women’s Affairs to develop and provide training on gender awareness, gender analysis, gender mainstreaming and issues in reproductive health and HIV/AIDS to staff of the Ministry of Health at all levels (Vong and Ros 2016, forthcoming).

## Discussion

We have applied a health systems gender analysis framework (Morgan *et al.* 2016) to analyse a range of different data sets in order to examine how gender shapes HRH in different fragile and post-conflict settings. This has enabled a holistic understanding of how gender plays out across multiple levels and how gender divisions of labour and caring responsibilities affect access to training and career choice, how gender norms and stereotypes shape who does what and levels of seniority, and how this in turn is shaped by policy and practice norms within different contexts. The studies also highlight how gender intersects both life cycle stages and other axes of inequity such as age, poverty or marital status to shape experience, choices made and room for manoeuvre (e.g. whether to continue working on limited or no pay and survival strategies during conflict). Gender intersects also with professional hierarchies, with medicine often being equated with leadership, limiting in some contexts (e.g. Zimbabwe) women’s potential for progression.

It is well established that occupational roles within the health workforce are highly gendered and health systems rely on a foundation of health workers that are often informal, poorly paid or not paid at all, poorly supported and disproportionately female (George 2008). Men typically cluster in more highly paid medical roles and women in less prestigious but crucial mid- and low-level caring nursing and support roles. In some OECD countries, according to the World Health Organisation, women represent over 90% of nursing and midwifery personnel.^5^ The post-conflict and post-crisis countries included in this study share these patterns of gender bias in terms of how does what. Similarly, the findings on gender asymmetries (penalizing women) with respect to further training and differential remuneration are in line with wider literature (George 2008, Newman 2014).

Gender relations and identities are inherently unstable and dynamic and interact with social change processes in all contexts (Tolhurst *et al.* 2012). Gender relations are constantly changing and gendered meanings of health occupations are constantly renegotiated as the health workforce change. Our analysis shows that this change may be intensified in times of conflict and fragility, bringing both challenges and opportunities. For example, staff in many areas have had to take on additional responsibilities beyond their formal roles to cover for staffing gaps and an exodus during crises. These allow, for example, mid-level female cadres to assume additional responsibilities and extend their skills, though these may come at some cost in terms of domestic responsibilities. However, over time as more formal training and deployment occurs, these cohorts can find themselves without the qualifications which are now needed for promotion and recognition (as occurred in Zimbabwe, where elderly women with great skills and experience were still on junior grades in formal terms) (Chirwa *et al.* 2016a). Our in-depth interviews with health workers in conflict-affected areas suggested that the workforces who lived through conflict and continued to work are very female-dominated. This is compatible with the wider health sector statistics, but remains surprising, given the insecurity in the region. Women showed special resilience and courage in staying in areas where physical threats were an everyday risk and reality, supported by links to families and communities. During the conflict in northern Uganda, health workers displayed values like empathy, professionalism and selflessness. This is something to be celebrated, rewarded and reinforced after the conflict (Namakula and Witter 2014a, b).

During the conflict, gender relations influence health workers’ decisions to enter the health workforce, upgrade skills, and remain in or leave the workforce, and the range of coping strategies deployed. Gender segregation by occupational roles, under-staffing in the remote areas and lack of responsiveness to life course events for workers with family responsibilities play a role in limiting access to training and promotion for women in particular. This was also found in other studies in Zambia and Uganda (Newman 2014).

The gender-blind HRH incentive policies and other broader national health policies fail to recognize the need to reward and support cohorts which have maintained services—at often great personal cost—through crisis periods, and also neglect the importance of breaking down professional silos. Just as women need support to train and work as doctors, so male nurses need support and encouragement. More involvement of the female and male staff of different cadres in the policy process and in assessing the impact of policies would also produce more effective and equitable health systems. In addition, many of the imbalances are rooted in gender norms and relations within families and communities, which can only be addressed over time and through dialogue and exposure to new ideas. This requires the health sector to build relationships with other sectors to promote action and change.

The strengths of our approach is the application of a recently developed gender analysis framework and the deployment of multiple methods (including those which allow analysis of gendered experiences through time) across different fragile and conflict affected contexts. The researchers undertaking the research are all embedded within their respective contexts, bringing their own tacit knowledge on gendered experiences and institutional positionality to the analytical process (Ritchie and Lewis 2004). Although there was some variation in use of methods in different contexts (additional qualitative research was undertaken in Cambodia and Zimbabwe, for example), the synthesis of findings from multiple contexts and methods has enabled a robust holistic analysis of how gender relations shapes the multiple experiences of health workers in fragile and conflict affected contexts. A weakness is that our analysis did not extend to close to community providers such as community health workers, who are arguably a particularly critical cadre in fragile and conflict affected contexts.

Our findings echo Schofield’s assertion that gender is a strong dynamic in the pattern of work forces (Schofield 2015). They also illustrate additional vulnerabilities of health workers during conflict and crisis (Witter *et al.* 2017a). Unless these are proactively addressed during the post-crisis reconstruction, health workforces will remain too few, poorly distributed and unable to meet the health needs of vulnerable populations. Practical steps need to be taken to identify gender barriers proactively and engage staff on ways of addressing them. For example, barriers to training and gender norms for specific cadres can be tackled, supporting all staff to realize their potential. Family-friendly working practices are important in all settings, but insecure areas need specific measures to ensure all staff can feel safe. Career paths can be re-engineered to allow leadership development for a wider range of professionals.

## Conclusion

This article presents empirical research across multiple fragile and conflict-affected contexts to assess how gender shapes human resource for health contexts. Addressing gender inequities in human resources is critical in all contexts if SDGS are to be realized. Our analysis demonstrates the opportunities and constraints in the post-conflict period, with new actors and a disruption to previous settlements. This arguably constitutes a window through which to rethink delivery and develop strategies to build more gender equitable health systems, although evidence shows that the timing of this window may vary (Bertone *et al.* 2014, Witter *et al.* 2017b). Underlying systems to support and maintain the functionality of the HRH system can be exceptionally weak at this stage, however, and governance challenged by multiple actors and the aftermath of conflict and crisis. However, there may be opportunities to recognize and address gender-related barriers; and these need to be seized to promote gender equity and ultimately more resilient, responsive and equitable health systems.

## Footnotes

1
http://resyst.lshtm.ac.uk/rings.

2 Department of Human Resources, MoH (2011), as cited in Men *et al*. (2011).

3 Referring to a handmade piece of cloth specially designed to carry babies on the back. This is still used by many women of lower socio-economic class.

4
http://www.buildingbackbetter.org/case-studies-2/sierra-leone.

5
http://www.who.int/hrh/statistics/spotlight_2.pdf?ua=1.
